# To Teach Doctors How to Cure Disease without Drugs

**Published:** 1911-02

**Authors:** 


### To Teach Doctors How to Cure Disease Without Drugs.

Temple University, of Philadelphia, has inaugurated a course of medical training said to be unique among the medical schools of the world. The new course has to do with methods of effecting cures and developing the body to its highest efficiency without the use of drugs. Dr. J. Madison Taylor has been appointed adjunct professor to take charge of the new department. He has just returned from an extensive tour abroad, from which he brings much instructive data for his new course of lectures to Temple medical students.

